# A Novel and Major Quantitative Trait Locus for Fusarium Crown Rot Resistance in a Genotype of Wild Barley (*Hordeum spontaneum* L.)

**DOI:** 10.1371/journal.pone.0058040

**Published:** 2013-03-11

**Authors:** Guangdeng Chen, Yaxi Liu, Jun Ma, Zhi Zheng, Yuming Wei, C. Lynne McIntyre, You-Liang Zheng, Chunji Liu

**Affiliations:** 1 Commonwealth Scientific and Industrial Research Organisation (CSIRO) Plant Industry, St Lucia, Queensland, Australia; 2 Triticeae Research Institute, Sichuan Agricultural University, Wenjiang, Chengdu, China; 3 School of Plant Biology, The University of Western Australia, Perth, Western Australia, Australia; China Agricultural University, China

## Abstract

Fusarium crown rot (FCR), caused by various *Fusarium* species, is a destructive disease of cereal crops in semiarid regions worldwide. As part of our contribution to the development of Fusarium resistant cultivars, we identified several novel sources of resistance by systematically assessing barley genotypes representing different geographical origins and plant types. One of these sources of resistance was investigated in this study by generating and analysing two populations of recombinant inbred lines. A major locus conferring FCR resistance, designated as *Qcrs.cpi-4H,* was detected in one of the populations (mapping population) and the effects of the QTL was confirmed in the other population. The QTL was mapped to the distal end of chromosome arm 4HL and it is effective against both of the *Fusarium* isolates tested, one *F. pseudograminearum* and the other *F. graminearum*. The QTL explains up to 45.3% of the phenotypic variance. As distinct from an earlier report which demonstrated co-locations of loci conferring FCR resistance and plant height in barley, a correlation between these two traits was not detected in the mapping population. However, as observed in a screen of random genotypes, an association between FCR resistance and plant growth rate was detected and a QTL controlling the latter was detected near the *Qcrs.cpi-4H* locus in the mapping population. Existing data indicate that, although growth rate may affect FCR resistance, different genes at this locus are likely involved in controlling these two traits.

## Introduction

Fusarium crown rot (FCR) is a chronic and severe disease of cereals in many parts of the semiarid regions of the world [1]. The economic importance of FCR stems mainly from yield losses but glasshouse-based assays showed that FCR-infected wheat plants could also produce mycotoxins in grains as well as other tissues [2]. Data from the Pacific Northwest of USA showed that the disease could reduce yields of malting barley cultivars by an average of 13% [3]. Recent surveys in Australia showed that FCR can be found in every wheat and barley growing region [1] and causes an estimated annual yield loss of some $97 million Australian dollars between wheat and barley [4], [5].

Field surveys in Queensland and New South Wales of Australia found *F. pseudograminearum* is the most prevalent pathogen for FCR but the disease can be caused by many different species of *Fusarium* [6]. Various practices have been assessed for their effectiveness in managing levels of FCR. These include crop rotation and stubble burning to reduce inoculum load [7], [8]. These practices have apparently not been very effective as the incidence of FCR has increased in Australia as well as in many other cereal growing regions worldwide in recent years, most likely due to the high intensity of cereals in cropping systems combined with the wider adoption of minimum tillage for moisture conservation as FCR pathogens are carried over in residues [1], [3], [9]. It is well known that, compared with wheat, barley cultivars tend to show more severe FCR symptom but suffer less yield loss [3]. Similar to the difference in visual symptoms between these crops, barley plants accumulate much higher concentrations of *Fusarium* pathogens than wheat plants at every stage of FCR infection [10]. Thus, growing resistant barley cultivars could not only reduce yield loss of the barley crop itself but also yield loss in barley or other cereal crops in the following years by reducing the inoculum load.

Effective breeding of resistant cultivars requires quality sources of resistance and understanding the genetics of a few high quality resistance sources can further enhance breeding efficiency. Significant progresses in identifying QTL conferring FCR resistance have been made recently in wheat [11]–[13]. However, similar studies in barley have been limited. The only report to date on mapping FCR resistance in barley was based on a population developed for studying non-FCR related traits and the single resistance locus on 3HL identified from this population co-locates with a major locus conferring plant height (PH) [14]. Recent results from wheat studies showed that PH can have a significant effect on FCR assessment [15]. Thus the effectiveness of the FCR QTL on 3HL needs to be further assessed by determining whether different genes are involved in conferring both height and FCR resistance at the locus concerned.

As part of our contribution to the development of FCR resistant cultivars, several genotypes with high levels of resistance were identified from an assessment of over 1,000 barley genotypes [16]. The genetics of FCR resistance in one of these best sources of resistance, AWCS276, was investigated by QTL mapping and the possible effects of PH and growth rate on FCR resistance assessed. Results obtained from these experiments are reported in this paper.

## Materials and Methods

### Plant Materials

The genotype AWCS276 was used as the source of FCR resistance in this study. This genotype, belonging to *Hordeum spontaneum* (L.), was one of the most resistant genotypes identified from a screen of 1,047 genotypes representing different geographical origins and plant types [16]. Two populations of F_7∶8_ recombinant inbred lines (RILs) were developed between AWCS276 and two different Australian cultivars in glasshouses at the Queensland Bioscience Precinct (QBP) in Brisbane, Australia. The first population, consisting of 132 lines of Baudin/AWCS276, was used for QTL mapping (mapping population). The second population, consisting of 131 lines of Fleet/AWCS276, was used to validate putative QTL identified in the mapping population.

### Evaluation of Resistance to FCR

Two *Fusarium* isolates, one *F. pseudograminearum* (CS3096) and one *F. graminearum* (CS3005), were used for FCR assessment. Both isolates were collected in northern New South Wales, Australia and maintained in the CSIRO collection [6]. The procedures used for inoculum preparation, inoculation and FCR assessment were based on that described by Li *et al.* [17]. Specifically, inoculum was prepared using plates of ½ strength potato dextrose agar. Inoculated plates were kept for 12 days at room temperature (about 22°C constant) before the mycelium on the plates were scraped. The plates were then incubated for a further 7–12 days under a combination of cool white and black fluorescent lights with 12-hour photoperiod. The spores were then harvested and the concentration of spore suspension was adjusted to 1×10^6^ spores/ml. Tween 20 was added (0.1% v/v) to the spore suspension prior to use.

Seeds were germinated in Petri dishes on three layers of filter paper saturated with water. The germinated seedlings were immersed in the spore suspension for 1 min and two seedlings were planted into a 5 cm square punnet (Rite Grow Kwik Pots, Garden City Plastics, Australia) containing sterilized University of California mix C (50% sand and 50% peat v/v). The punnets were arranged in a randomized block design in either a glasshouse or a controlled environment facility (CEF). Settings for the glasshouse were: 25/18 (±1)°C day/night temperature and 65/80 (±5)% day/night relative humidity, with natural sunlight levels and variable photoperiod depending on the time of year. The settings for the CEF were: 25/16 (±1)°C day/night temperature and 65/85% day/night relative humidity, and a 14-hour photoperiod with 500 µmol m^-2^s^-1^ photon flux density at the level of the plant canopy. To promote FCR development, water-stress was applied during the FCR assessment. Inoculated seedlings were watered only when wilt symptoms appeared.

For QTL mapping, four replicated trials were carried out using the *F. pseudograminearum* isolates CS3096 (designated as CR96-CEF01, CR96-CEF02, CR96-GH01, CR96-GH02, respectively) and two replicated trials were carried out using the *F. graminearum* isolate CS3005 (designated as CR05-CEF01 and CR05-CEF02, respectively). For QTL validation in the second population, two replicated trials were conducted, one in the glasshouse and the other in the CEF. Both trials were conducted using the *F. pseudograminearum* isolate CS3096. Each of the eight trials contained two replicates, each replicate with ten seedlings. FCR severity was assessed 21 days after inoculation, using a 0 (no obvious symptom) –5 (whole plant severely to completely necrotic) scale as described by Li *et al.* [17]. A disease index (DI) was then calculated for each line following the formula of DI = (Σ_nX_/5N)×100, where X is the scale value of each plant, n is the number of plants in the category, and N is the total number of plants assessed for each line.

### Evaluation of Plant Height and Heading Date

To assess possible effects of PH and heading date (HD) on FCR resistance, three trials were conducted using the RIL population of Baudin/AWCS276. One of these was a pot-based glasshouse trial at the QBP while the other two were field trials conducted at the CSIRO Research Station at Gatton in Queensland (27°34′S, 152°20′E). The pot trial consisted of two replicates. Three plants, each in a different pot of 2.0 L, were used in each of the replicates. Measurements for each of the traits were obtained from the two tallest tillers for each plant and their averages were used for statistical analysis. The two field trials conducted were sown in June 2011 and June 2012, respectively. Randomized blocks design was used for both of the field trials, each with three replicates. For each replicate, 20 seeds for each of the RILF_8_ lines were sown in a single 1.5 metre row with a 25 cm row-spacing. Six measurements were obtained for each trait from the six tallest tillers in each row and the average from the six measurements was used for statistical analyses. The HD of a line was recorded as the day on which about 50% of the spikes emerged from first tillers.

### Molecular Marker Analysis

The methods used for DNA isolation and marker analysis were as described by Chen *et al.* [18]. PCR-based markers of SSR (simple sequence repeat) and CAPS (cleaved amplified polymorphic sequences) were used in this study. These markers were selected based on their locations in existing barley linkage maps [19]–[21] so that the whole genome was covered with a distance of about 15 cM between two adjacent markers. PCR reactions for the amplification of markers were carried out in a total volume of 12 µl containing 25 ng genomic DNA, 0.2 µM of forward and reverse primer, 3 mM MgCl_2_, 0.2 mM dNTPs and 0.5 U Taq DNA polymerase. During PCR reactions the marker products were labeled with α-[^33^P]dCTP (3,000 ci/mmol). PCR amplification was conducted using a Gene Amp PCR System 2700 thermocycler (PE Applied Biosystems, Foster City, Calif.) programmed with the cycling conditions: one cycle of 3 min at 94°C, 35 cycles of 1 min at 94°C, 1 min at the appropriate annealing temperature (ranging from 50°C to 56°C depending on the primers) and 1 min at 72°C, with a final extension step of 5 min at 72°C. The amplified products were mixed with an equal volume of loading dye, denatured at 95°C for 5 min, and 3.8 µl samples was run on a denaturing 5% polyacrylamide (20∶1) gel at 90 W for 2 hrs. The gels were subsequently dried using a gel dryer for 30 min at 80°C and exposed to Kodak X-Omat X-ray film for 4–6 days. The markers were firstly tested against Baudin and AWCS276. Polymorphic markers identified were then used to analyse the whole population of Baudin/AWCS276.

### Data Analysis and QTL Mapping

Statistical analyses were performed using GenStat for Windows, 13th edition (copyright Lawes Agricultural Trust, Rothamsted Experimental Station, UK) and the SPSS statistics 17.0 for Windows statistical software package (SPSS Inc., Chicago, IL). For each trial, the following model of mixed-effects was used: Yij = *µ*+*ri*+*gj*+*wij*, where: Y*ij* = trait value on the *j*th genotype in the *i*th replication; *µ* = general mean; *ri* = effect due to *i*th replication; *gj* = effect due to the *j*th genotype; *wij* = error or genotype by replication interaction, where genotype was treated as a fixed effect and that of replicate as random. The effects of replicate and genotype for each trait were determined using ANOVA. The Pearson correlation coefficient was estimated between traits and trials.

Segregation ratios of the markers assessed were tested by Chi-square goodness-of-fit to a 1∶1 ratio at the significance level of *p = *0.01. Linkage analysis was carried out using the computer package JoinMap [22]. LOD thresholds from 3 to 10 were tested, until a threshold with the optimum number of markers in linkage groups maintaining linkage order and distance was obtained. The Kosambi mapping function was used to convert recombination ratios to map distances.

MapQTL® 5.0 [23] was used for QTL analysis. The Kruskal–Wallis test was used in a preliminary testing of associations between markers and FCR resistance. Interval mapping (IM) was then used to identify major QTL. Automatic cofactor selection was used to fit the multiple QTL model (MQM) and to select significantly associated markers as cofactors. For each trial, a test of 1,000 permutations was performed to identify the LOD threshold corresponding to a genome-wide false discovery rate of 5% (*P*<0.05). Based on the permutation test, a threshold LOD value was used to declare the presence of a QTL. A linkage map showing the QTL positions was drawn using MAPCHART [24].

## Results

### Characterization of FCR Reaction in the Population of Baudin/AWCS276

**Figure 1:**
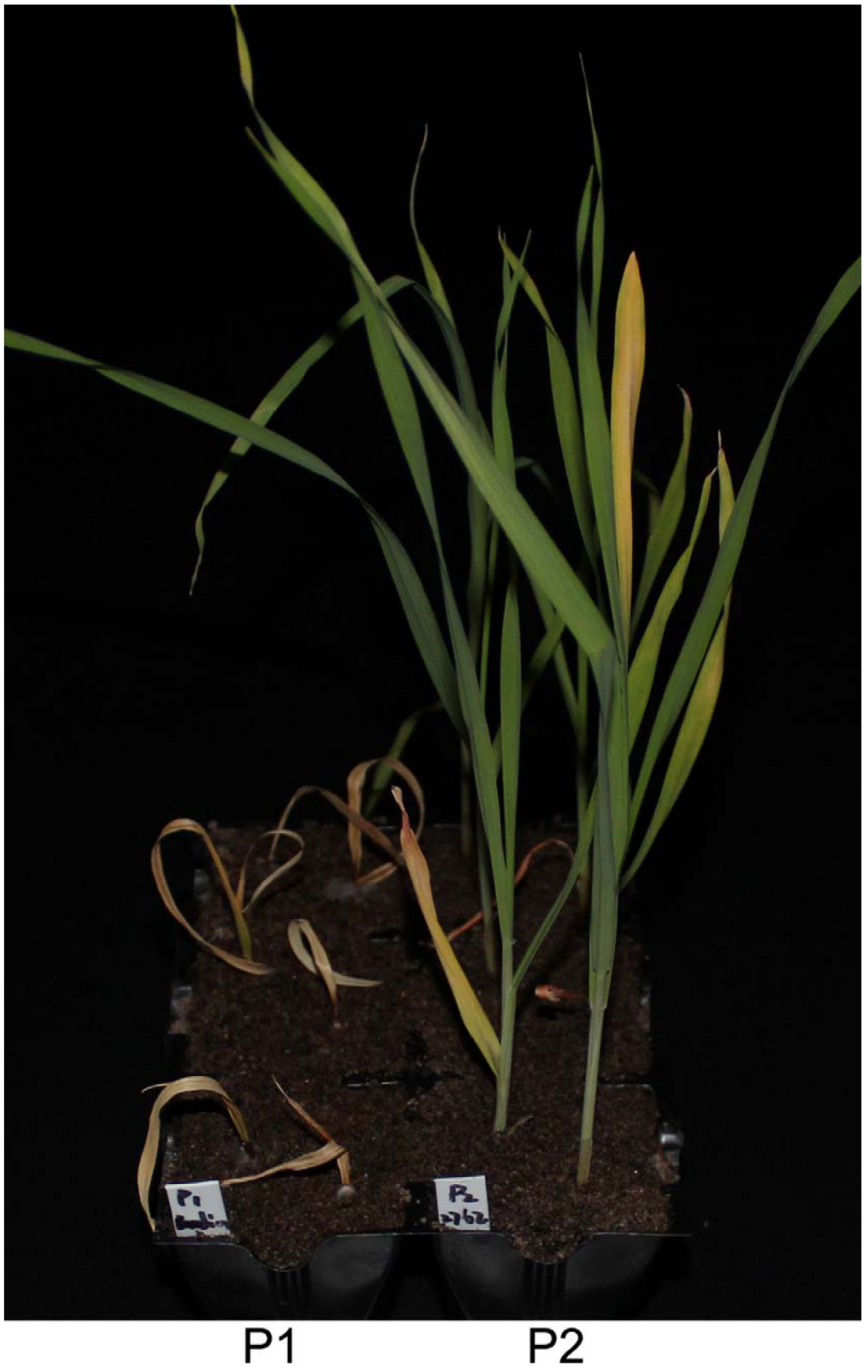
Difference in resistance to Fusarium crown rot between Baudin (P1) and AWCS276 (P2) detected with an isolate of *F. pseudograminearum* (CS3096). The photo was taken 21 days post inoculation.

Drastic differences in FCR resistance were detected between the commercial cultivar Baudin and the source of resistance AWCS276 using either the *F. graminearum* or the *F. pseudograminearum* isolate analysed (Figure 1). Among the six trials conducted, DI values for AWCS276 ranged between 10.0 and 28.0 (average 17.5) and those for Baudin ranged between 90.0 and 98.0 (average 94.2). Lines with better FCR resistance than the resistant parent AWCS276 were detected in five of the six trials. Differences in FCR resistnace between the best lines from the two CEF trials with the use of the *F. pseudograminearum* isolate and the resistant parent were more than 50% (Table 1). DI values were significantly and positively correlated among the six trials, with correlation coefficients (*R*) ranging from 0.50 to 0.82 (Table 2).

**Table 1:** Disease index of FCR severity in the population of Baudin/AWCS276.

Trial^a^	Parent		Population			
	Baudin	AWCS276	Min	Max	Mean	SD
CR96-CEF01	95.0	21.5	10.0	86.0	48.8	21.15
CR96-CEF02	98.0	28.0	13.5	91.0	53.2	22.78
CR96-GH01	90.0	12.0	16.0	89.0	43.2	16.56
CR96-GH02	93.5	10.0	7.5	82.5	37.7	17.16
CR05-CEF01	98.0	18.0	14.5	89.0	46.5	17.90
CR05-CEF02	90.5	15.5	12.0	94.0	47.6	18.48

a The two trials conducted using the *F. pseudograminearum* isolate CS3096 in controlled environment facilities (CEF) were designated as CR96-CEF01 and CR96-CEF02, respectively; the two trials conducted using the *F. pseudograminearum* isolate CS3096 in glasshouses were designated as CR96-GH01 and CR96-GH02, respectively; and the two trials conducted using the *F. graminearum* isolate CS3005 in CEF were designated as CR05-CEF01 and CR05-CEF02, respectively.

**Table 2:** Correlation coefficients of FCR resistance in the population of Baudin/AWCS276 among six replicated trials.

Trial^a^	CR96-CEF01	CR96-CEF02	CR96-GH01	CR96-GH02	CR05-CEF01	CR05-CEF02
CR96-CEF01	1.00					
CR96-CEF02	0.82**	1.00				
CR96-GH01	0.62**	0.72**	1.00			
CR96-GH02	0.67**	0.74**	0.69**	1.00		
CR05-CEF01	0.50**	0.63**	0.56**	0.60**	1.00	
CR05-CEF02	0.56**	0.62**	0.61**	0.60**	0.81**	1.00

a The two trials conducted using the *F. pseudograminearum* isolate CS3096 in controlled environment facilities (CEF) were designated as CR96-CEF01 and CR96-CEF02, respectively; the two trials conducted using the *F. pseudograminearum* isolate CS3096 in glasshouses were designated as CR96-CEF01 and CR96-CEF02, respectively; and the two trials conducted using the *F. graminearum* isolate CS3005 in CEF were designated as CR05-CEF01 and CR05-CEF02, respectively; ‘**’ significant at *p*<0.01.

### Linkage Map Construction and QTL Analysis

**Figure 2:**
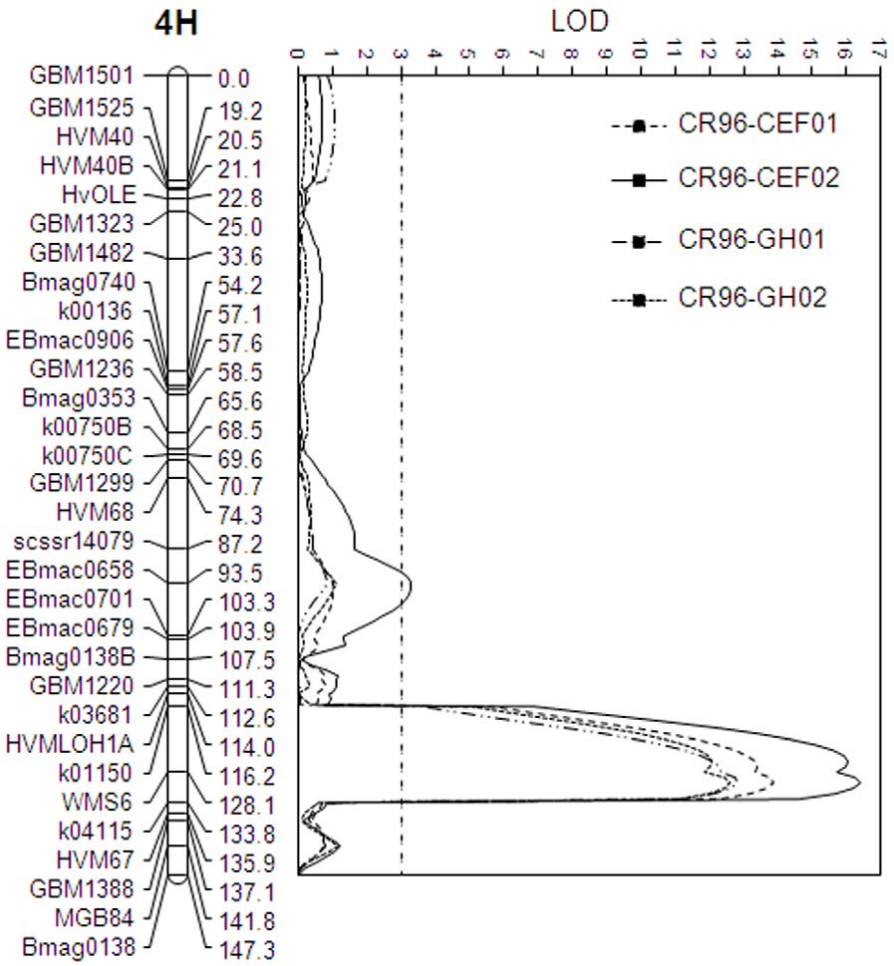
QTL conferring FCR resistance detected in the population of Baudin/AWCS276 using an isolate of *F. pseudograminearum* (CS3096). Marker positions are shown to the left of the linkage map and distances in centiMorgan (cM) between loci are shown to the right. The vertical dotted line indicates the significance threshold of LOD 3.0.

Of the markers assessed, 111 detected difference between Baudin and AWCS276. These markers formed 15 linkage groups, covering a total genetic distance of 904.8 cM. The partial genetic linkage map was exploited for initial QTL detection using single marker analysis. This analysis detected a single QTL conferring FCR resistance on chromosome arm 4HL. We have designated the QTL as *Qcrs.cpi-4H*, where ‘crs’ represents ‘Fusarium crown rot severity’ and ‘cpi’, CSIRO Plant Industry following convention. Additional markers on this chromosome were then assessed against the two parents. These included 19 SSR and 16 CAPS markers. Of these, 14 were polymorphic between the two parental genotypes and they detected a total of 16 loci. Together with the 14 markers in the initial linkage map obtained, the 28 markers on this chromosome detected a total of 31 loci. Segregation distortion was detected for none of these loci (Figure 2).

**Figure 3:**
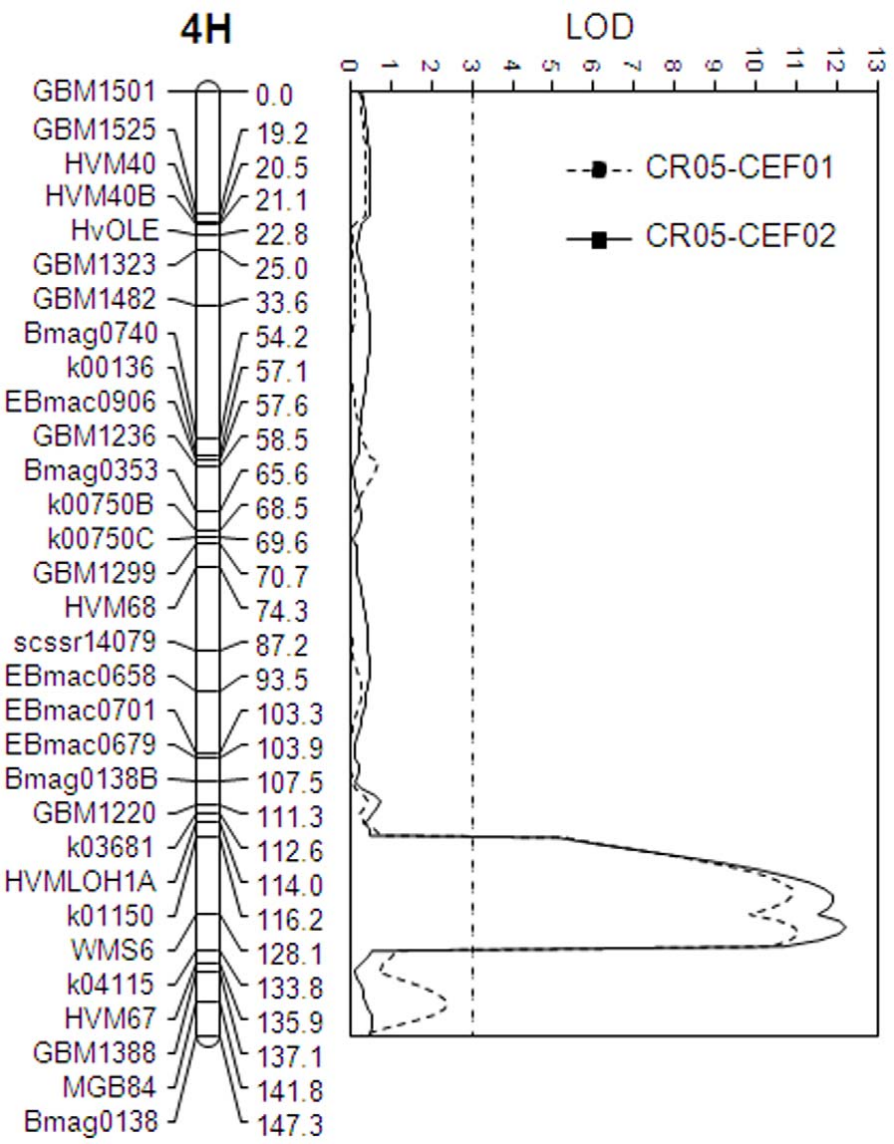
QTL conferring FCR resistance detected in the population of Baudin/AWCS276 using an isolate of *F. graminearum* (CS3005). Marker positions are shown to the left of the linkage map and distances in centiMorgan (cM) between loci are shown to the right. The vertical dotted line indicates the significance threshold of LOD 3.0.

QTL mapping based on the improved linkage map of 31 loci found that *Qcrs.cpi-4H* explained between 39.3% and 45.3% of the phenotypic variation in the four trials conducted using the *F. pseudograminearum* isolate CS3096 (Table 3), and the QTL explained 42.8% and 40.8% of the phenotypic variation, respectively, in the two trials conducted using the *F. graminearum* isolate CS3005 (Table 3). Based on MQM analysis, markers flanking the *Qcrs.cpi-4H* locus were K01150 and WMS6 and the marker mapping most closely to the peak of the QTL was WMS6 (Figures 2 and 3).

**Table 3:** QTL for FCR severity identified in the population of Baudin/AWCS276.

Trial^a^	Analysis^b^	Flanking markers	LOD	R^2^(%)
CR96-CEF01	IM	GBM1220 & Bmag0138	13.6	40.1
	MQM	K01150 & WMS6	13.9	39.2
	IM	EBmac0658 & EBmac0701	3.7	15.1
CR96-CEF02	IM	GBM1220 & Bmag0138	16.0	45.3
	MQM	K01150 & WMS6	16.4	44.6
	IM	EBmac0658 & EBmac0679	6.4	23.8
	MQM	EBmac0658 & EBmac0701	3.3	6.6
CR96-GH01	IM	GBM1220 & Bmag0138	12.7	39.3
	MQM	K01150 & WMS6	12.8	37.0
CR96-GH02	IM	GBM1220 & MGB84	12.7	39.5
	MQM	K01150 & WMS6	12.6	37.0
	IM	EBmac0658 & EBmac0701	3.4	13.2
CR05-CEF01	IM	GBM1220 & Bmag0138	11.3	42.8
	MQM	K01150 & WMS6	11.0	35.9
CR05-CEF02	IM	GBM1220 & Bmag0138	12.5	40.8
	MQM	K01150 & WMS6	12.3	37.1

a The two trials conducted using the *F. pseudograminearum* isolate CS3096 in controlled environment facilities (CEF) were designated as CR96-CEF01 and CR96-CEF02, respectively; the two trials conducted using the *F. pseudograminearum* isolate CS3096 in glasshouses were designated as CR96-CEF01 and CR96-CEF02, respectively; and the two trials conducted using the *F. graminearum* isolate CS3005 in CEF were designated as CR05-CEF01 and CR05-CEF02, respectively;

b IM = analysis conducted using interval mapping and MQM = analysis conducted using the multiple QTL model.

A second QTL, closely linked and proximally located to *Qcrs.cpi-4H*, was detected in three of the four trials conducted using the *F. pseudograminearum* isolate CS3096 based on IM analysis (Table 3). However, this QTL was significant in only one of these trials based on MQM analysis (Figure 2), with a LOD value of 3.3 and explaining 6.6% of the phenotypic variance (Table 3). As this QTL showed only limited effects and was not detected in all of the trials and it is known that QTL mapping does not provide adequate resolution for loci with a genetic distance of 20 cM or less [25], this minor FCR QTL was not considered further.

### Effects of Qcrs.cpi-4H in the Population of Fleet/AWCS276

Possible effects of *Qcrs.cpi-4H* were further assessed in a second population between Fleet and AWCS276. The most closely linked SSR marker identified from the population of Baudin/AWCS276, WMS6, was used to identify individual lines with alleles from either of the two parental genotypes. Based on the presence or absence of the allele from the resistant parent ‘AWCS276’, the RILF_7∶8_ lines were placed into two groups. DI values for those lines in the group with the AWCS276 allele varied between 6.0 to 84.0 with an average of 42.6 in the CEF trial and varied between 11.0 and 85.5 with an average of 43.7 in the glasshouse trial. The DI values for those lines in the group with the allele from the commercial cultivar Fleet varied between 27.0 and 97.0 with an average of 58.1 in the CEF trial and varied between 24.5 and 99.0 with an average of 61.5 in the glasshouse trial. The difference between the average DI values of the two groups of lines was 26.7% for the CEF trial and 28.9% for the glasshouse trial (Table 4).

**Table 4:** Effects of *Qcrs.cpi-4H* in the population of Fleet/AWCS276.

Trial^a^	RR	rr	Difference (%)	p value
CEF	42.6	58.1	26.7	<0.01
GH	43.7	61.5	28.9	<0.01

a ‘CEF’ and ‘GH’ represent ‘controlled environmental facilities’ and ‘glasshouse’, respectively; ‘RR’ and ‘rr’ represent homozygous allele from AWCS276 and the commercial cultivar Fleet, respectively.

### Effects of Plant Height and Heading Date on FCR Resistance

For estimating possible effects of PH and growth rate on FCR severity, DI values of the six FCR trials for each line of the mapping population were used in correlation analyses. Significant correlations between FCR resistance and PH were not detected using PH data collected from either of the field or the glasshouse trial (Table 5). QTL analysis detected a single locus for PH from each of the three trials. Different from that of *Qcrs.cpi-4H*, the PH QTL locates on chromosome 3H (Table 6).

**Table 5:** QTL for plant height and heading date identified in the population of Baudin/AWCS276.

Trial^a^	QTL^b^	Chromosome	Flanking markers	LOD	R^2^(%)
PH-FD(2011)	IM	3H	Bmag0606 & Bmag0013	12.9	74.6
	MQM	3H	Bmag0606 & Bmag0013	11.5	64.1
PH-FD(2012)	IM	3H	Bmag0606 & GBM1462B	11.5	61.3
	MQM	3H	Bmag0606 & Bmag0013	10.8	59.4
PH-GH(2011)	IM	3H	Bmag0606 & GBM1462B	10.7	62.1
	MQM	3H	Bmag0606 & Bmag0013	10.5	55.8
HD-FD(2011)	IM	4H	GBM1388 & MGB84	3.0	12.3
	MQM	4H	GBM1388 & MGB84	3.0	11.4
HD-FD(2012)	IM	4H	GBM1388 & MGB84	3.6	12.8
	MQM	4H	GBM1388 & MGB84	3.6	12.1
HD-GH(2011)	IM	4H	HVM67 & MGB84	5.3	20.0
	MQM	4H	GBM1388 & MGB84	5.2	18.1

a ‘PH’ and ‘HD’ represent ‘plant height’ and ‘heading date’, respectively; ‘FD’ and ‘GH’ indicate ‘field trial’ and ‘glasshouse trial’, respectively;

b IM = analysis conducted using interval mapping; MQM = analysis conducted using the multiple QTL model.

**Table 6:** Correlation coefficients between FCR severity, plant height and heading date among six FCR trials in the Baudin/AWCS276 population.

Trial^a^	CR96-CEF01	CR96-CEF02	CR96-GH01	CR96-GH02	CR05-CEF01	CR05-CEF02
PH-FD(2011)	0.09	0.12	0.09	0.03	0.15	0.12
PH-FD(2012)	0.09	0.10	0.12	0.07	0.12	0.12
PH-GH(2011)	0.02	0.04	−0.02	−0.06	0.09	0.15
HD-FD(2011)	−0.29**	−0.35**	−0.29**	−0.27**	−0.32**	−0.32**
HD-FD(2012)	−0.32**	−0.32**	−0.30**	−0.32**	−0.34**	−0.36**
HD-GH(2011)	−0.29**	−0.34**	−0.34**	−0.30**	−0.39**	−0.38**

a The two trials conducted using the *F. pseudograminearum* isolate CS3096 in controlled environment facilities (CEF) were designated as CR96-CEF01 and CR96-CEF02, respectively; the two trials conducted using the *F. pseudograminearum* isolate CS3096 in glasshouses were designated as CR96-CEF01 and CR96-CEF02, respectively; and the two trials conducted using the *F. graminearum* isolate CS3005 in CEF were designated as CR05-CEF01 and CR05-CEF02, respectively; ‘PH’ and ‘HD’ represent ‘plant height’ and ‘heading date’, respectively; and ‘FD’ and ‘GH’ indicate the ‘field trial’ and ‘glasshouse trial’, respectively; ‘**’ significant at *p*<0.01.

**Figure 4:**
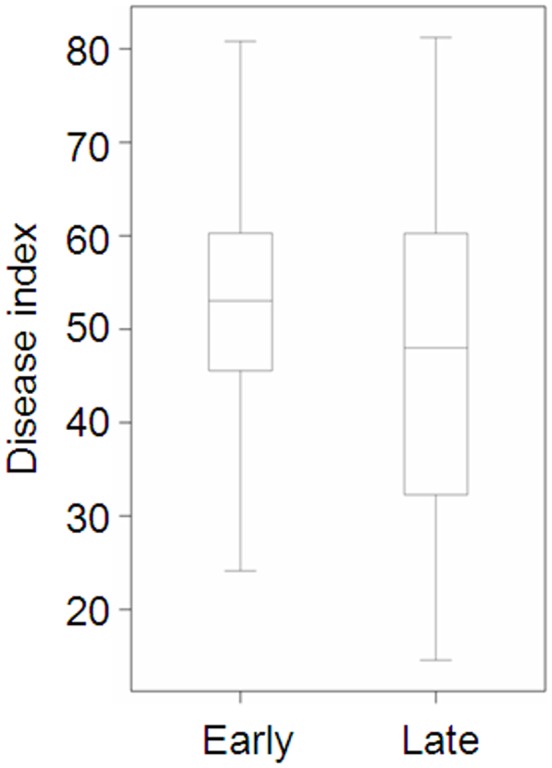
Distributions and medium of Fusarium crown rot disease index among genotypes in each of the two groups with different growth rates.

Significant correlations were detected between HD and FCR resistance (Table 6). When the lines of the mapping population were placed into either a ‘Early heading’ and a ’Late heading’ group based on their HD, the average DI value of the ‘Late heading’ group was significantly lower than that of the ‘Early heading’ group. The top 10 lines (7.6%) of the population all belong to the ‘Late heading’ group. However, a wide range of DI values was found for lines in both groups. FCR severity for some of the lines in the ‘Early heading’ group was also low and lines with severe FCR infection were also found in the ‘Late heading’ group (Figure 4).

QTL analysis detected a locus conferring HD using data collected from either the field trials or the glasshouse trial. The QTL was mapped closely to *Qcrs.cpi-4H* and it explained 12.3%, 12.8% and 20.0% of the phenotypic variance in the field and glasshouse trials, respectively (Table 5).

## Discussion

We report in this paper a QTL study on FCR resistance in one of the most resistant genotypes obtained from a systematic screening in barley [16]. A major QTL, located at the distal end of chromosome 4HL, was detected in this wild barley genotype belonging to *H. spontanneum* (L.). The QTL was detected in both of the populations analysed and it explains up to 45.3% of the phenotypic variance in both glasshouse and field trials. The QTL was effective against both of the *Fusarium* isolates analysed, one *F. pseudograminearum* and one *F. graminearum*. These results are in agreement with those obtained in wheat [11], [12] and it seems that FCR resistance in neither of these species is *Fusarium* species specific.

Colinearity between genomes of closely related species has been used to predict and locate genes and markers of interest. Apart from a few well characterized translocations between the A and B genomes of wheat [26], gene order between barley and wheat are highly conserved [27]. Considering the existence of the 4AL/5AL/7BS translocations in wheat, two of the three homoeoloci of the *Qcrs.cpi-4H* locus in wheat should be found on the long arms of chromosomes 4B and 4D, respectively. The location of the third homoeolocus, however, could be on three possible chromosomal regions. If it was not involved in the 4AL/5AL translocation, the third locus should be on the current short arm of chromosome 4A. However, if the locus was involved in the translocation, the homoeolocus should be located on either the current long arm of chromosome 4A or the short arm of chromosome 7B [26]. One of the reported wheat FCR QTL does locate on chromosome 4B [28]. However, the location of the 4B FCR QTL is near the *Rht-B1b* locus [28] which is on the short arm of the chromosome. Thus the FCR QTL on 4B is unlikely to be homoeologous with *Qcrs.cpi-4H.*

Previous results show repeatedly that PH may affect FCR resistance. These include the first QTL study of FCR resistance in barley which was based on a population generated for non-FCR related traits. A single FCR QTL on the long arm of chromosome 3H was detected in that study and the FCR QTL co-located with a gene(s) conferring PH [14]. Similar effects of PH on FCR severity were also detected in wheat, using both segregating populations [12], [28] and near isogenic lines (NILs) for various *Rht* genes [15]. Such effects make it difficult to exploit those FCR resistance genes which locate closely to *Rht* loci. Fortunately, significant effects of PH on *Qcrs.cpi-4H* were not detected in the populations analysed in this study. The different results between these reports demonstrate that the association between height and FCR resistance is genotype dependent, which would facilitate the efforts of FCR breeding.

A strong correlation between FCR resistance and growth rate was detected in barley when non-vernalized barley seeds were sown in a region where vernalizaiton conditions do not exist, with late-heading genotypes giving better FCR resistance [16]. It is well known that growth rates are affected by many genes including those for vernalization, those for photoperiod response as well as those independent of vernalization and day-length response [29]. To further investigate the relationship between growth rate and FCR resistance, a QTL analysis was conducted for growth rate in the current study. A single QTL affecting growth rate was detected. Similar to that of *Qcrs.cpi-4H*, the QTL controlling growth rate was also located distally on the long arm of chromosome 4H. It is known that a gene controlling vernalization requirement resides at this chromosome region [30], [31]. The results obtained from the mapping population seem to be in agreement with that obtained in assessing random genotypes in that winter habit could contribute to better FCR resistance [16]. However, some of the early-heading lines segregated in both the mapping and the validation populations gave high levels of FCR resistance and some of the late-heading lines showed severe FCR symptom. Considering that only a single QTL was detected for each of these two traits in the mapping population used in this study and that QTL conferring FCR resistance was not located near genes controlling growth rate in the previous study [14], the available results indicated that FCR and growth rate are unlikely controlled by the same gene(s) thus breeding FCR resistant cultivars with different rates of development should be feasible.
